# Culture and Hallucinations: Overview and Future Directions

**DOI:** 10.1093/schbul/sbu012

**Published:** 2014-06-13

**Authors:** Frank Larøi, Tanya Marie Luhrmann, Vaughan Bell, William A. Christian, Smita Deshpande, Charles Fernyhough, Janis Jenkins, Angela Woods

**Affiliations:** ^1^Department of Psychology, University of Liège, Liège, Belgium;; ^2^Department of Anthropology, Stanford University, Stanford, CA;; ^3^King’s College London, Institute of Psychiatry, London, UK;; ^4^Department of Social Anthropology, Autonomous University of Barcelona, Bellaterra, Spain;; ^5^Department of Psychiatry and Addiction Services, Dr Ram Manohar Lohia Hospital, New Delhi, India;; ^6^Department of Psychology, Durham University, Durham, UK;; ^7^Department of Anthropology, University of California San Diego, San Diego, CA;; ^8^Centre for Medical Humanities, Durham University, Durham, UK

**Keywords:** hallucination, culture, ethnography, psychosis, religion

## Abstract

A number of studies have explored hallucinations as complex experiences involving interactions between psychological, biological, and environmental factors and mechanisms. Nevertheless, relatively little attention has focused on the role of culture in shaping hallucinations. This article reviews the published research, drawing on the expertise of both anthropologists and psychologists. We argue that the extant body of work suggests that culture does indeed have a significant impact on the experience, understanding, and labeling of hallucinations and that there may be important theoretical and clinical consequences of that observation. We find that culture can affect what is identified as a hallucination, that there are different patterns of hallucination among the clinical and nonclinical populations, that hallucinations are often culturally meaningful, that hallucinations occur at different rates in different settings; that culture affects the meaning and characteristics of hallucinations associated with psychosis, and that the cultural variations of psychotic hallucinations may have implications for the clinical outcome of those who struggle with psychosis. We conclude that a clinician should never assume that the mere report of what seems to be a hallucination is necessarily a symptom of pathology and that the patient’s cultural background needs to be taken into account when assessing and treating hallucinations.

## What Is Culture?

Anthropologists commonly use the term “culture” to describe shared patterns of meaning that are learned within a particular social world—“that complex whole which includes knowledge, belief, art, law, morals, custom, and any other capabilities and habits acquired by man as a member of society”^1^ or “patterns, explicit and implicit, of and for behaviour acquired and transmitted by symbols.”^2^ By the term, anthropologists draw attention to the fact that humans are meaning-making animals and that, over time, different groups of humans develop different habits in interpreting even the most basic features of their experience. The research reported here suggests that cultural expectations shape the way people pay attention to their sensory experience. These different patterns of attention may be responsible for differing experiences of hallucinations.

## Culture Can Affect What Is Identified as a Hallucination

One of the most significant factors in how culture affects the recognition of the experience of hallucination rests on the understanding of reality in the culture in question. Although there are many definitions used in the academic literature, many describe hallucinations as “false” perceptions. This definition can seem to depend on a specific understanding of reality alien to most humans, who accept some degree of supernatural reality.^3^

An ethnographic approach to hallucinations therefore becomes essential in understanding how members of particular societies identify and understand sensory events that would be recognized by secular observers as hallucinations and how they distinguish between unusual sensory events they regard as appropriate and those they identify as signs of illness. The richness of the ethnographic method captures meaning that experimental approaches will miss. For example, the Cashinahua, Siona, and Schuar peoples of the Upper Amazon all use the hallucinogenic brew ayahuasca as a spiritual guide. However, the Cashinahua consider the experiences as hallucinations that provide guidance,^4^ the Siona believe that ayahuasca provides access to an alternate reality,^5^ and the Schuar hold that all normal human experience is a hallucination and ayahuasca provides access to veridical reality.^6^ This is an important point because research on hallucinations usually involves asking people about experiences that are not explainable, have no obvious source or are not shared by others.^7^ Differing views of what constitutes veridical reality may affect how these experiences are reported. At the least, these cultural issues should shape the way researchers frame both their assessment methods and their research questions. More empirically, the fact that different cultural models of reality may lead to differing levels of reporting means that the kinds and rates of hallucinatory experience may vary between cultures in epidemiological studies due to different theories of the world and not just differing levels of experience.

## Different Patterns of Hallucinations

Both the ethnographic and clinical literatures agree that hallucinations are common in the nonclinical population.^8,9^ The form of hallucination in the clinical and nonclinical population are, however, relatively distinct, and there seem to be, broadly speaking, 3 dominant patterns.^10^

Persons with psychosis often hallucinate many times each day. These hallucinations may be unpleasant, even horrific. In the schizophrenia spectrum, hallucinations are primarily auditory, and they are often accompanied by strange, fixed beliefs (delusions) not shared by other people. It is also true that the voice-hearing experience of persons with psychosis is varied; Jenkins^11^ describes such a woman who did not consider hearing voices as “discontinuous with the self” but rather as “part of herself” and a struggle over moral goodness and “the right to be in the world.” It has been clear for many decades that serious psychotic disorder is recognized across cultures with a similar pattern of symptoms, despite increasing awareness that culture may shape the content, meaning, and possibly the severity of the symptoms.^12,13^

By contrast, hallucinations experienced in the general population are likely to be brief, not unpleasant and not experienced frequently.^14^ Depending on the way the question is asked, 10%–15% or more of the population report them.^15^ They are even more common among the bereaved. As many as 80% of those who have lost loved ones report seeing, hearing, or feeling the touch of the dead person even among Euro-American populations, in which speaking to the dead is not normative.^16^ Those with longer and happier marriages are more likely to report these sensory experiences, and for the most part, the experiences are comforting.^17^ An older study found an even higher rate (90%) among the Japanese,^18^ who at the time often maintained ties with the deceased through religious rituals. However, there are clearly cultural variations. The Achuar people of Ecuador prohibit remembrance practices and consider any form of reexperiencing of a specific person, including thoughts, visions, or dreams, as a threat to the soul of the experiencer. They do, however, seek sensory encounters with a dead person whose identity is obscure to them.^19^

Finally, there are also some people who have unusual sensory experiences as often as people who can be diagnosed with schizophrenia, yet without the intense distress psychosis carries in its wake, or any of its other symptoms—delusions, cognitive difficulties, or emotional flatness. Religious experts around the world also sometimes behave as if, and speak as if, they have frequent and ongoing hallucinatory experiences. We return to these experts below.

In addition, hallucinations may also arise as the result of the deliberate use of psychotropic agents such as ayahuasca or peyote. Religions incorporating such agents have been particularly common in the indigenous Americas, where shamans and other religious experts have sought visions and voices they take to be guidance from the spirit world.

## Hallucinations Are Often Culturally Meaningful

There is robust evidence that unusual sensory experiences have been given great importance as foundational spiritual experiences throughout the world—Moses and his burning bush, Paul on the road to Damascus, Arjuna’s vision of Krishna, Buddha beneath the Bo tree. Bourguignon^20^ examined data collected from the Human Relations Area File (HRAF) from 488 societies worldwide. In 62% of the cultures studied, hallucinations played a role in ordinary ritual practices. These hallucinations were positively valued, could be understood in the context of local beliefs and practices, and the presence of hallucinations was not usually associated with intake of psychoactive chemicals. Bourguignon thought that her rate was relatively low because the material in the HRAF was incomplete and the absence of a record of hallucinations in the archive did not imply the absence of the phenomenon from the society.

Typically, such sensory experiences of the immaterial are understood as contacts with gods, spirits, or the dead. While many such experiences never enter the historical record, others take on broad public meaning. Lourdes^21^ became a major healing shrine because a young girl, Bernadette Soubirous, reported that she saw the Virgin Mary there, and many people came to believe that indeed she had. The shrines of Fatima and Medjugore similarly draw millions of worshippers who believe that the Virgin appeared to specific individuals so that they saw her with their eyes and who come to worship and request favor from the Virgin at a place where her immaterial body was perceived with the physical human senses.

To become available as plausible experiences of the divine, such hallucinations must conform to local cultural expectations.^22^ The local population at Lourdes, expected Mary to act like a benign mother; had Bernadette reported seeing the blindingly powerful figure Mary was understood to be toward the end of the Middle Ages, the 19th-century French population would probably not have believed that she had seen the Virgin. At the same time, in each vision locale, a kind of fluid and evolving microculture develops, in which some features partake of a broader pattern—known through literature, visual media, and shared pilgrims—but others are idiosyncratic and innovative. At Lourdes, Bernadette behaved oddly, scratching up the earth to find the spring that would later become the focal point of pilgrimage. Taves^23^ similarly demonstrates that as the 19th century progressed, the capacity to hear God or the dead speak became more acceptable for ordinary Christians as spiritualism became a popular movement and began to change the way people thought about the human psyche. The same holds true in the way people become identified as religious experts. For example, the shaman-to-be usually must report certain kinds of phenomena that are understood by his or her broader social world to be the appropriate signs of the spirit. For example, among an Amazonian people called the Bororo, the novice shaman is identified when he has a dream of soaring high above the earth, like a vulture, and seeing the fiery cloud of smoke that indicates an attacking illness.^24^ Then, he must see a stone or anthill move, and he must hear a voice, when alone in the forest, that asks him where he is going.

In a social setting where hallucinations are taken as evidence of the supernatural or divine, people typically take considerable care to distinguish explicitly between the hallucinations of madness and hallucinations that indicate contact with the spiritual world. When someone’s experience matches cultural expectations, this is often taken to demonstrate that the unusual sensory experience is of the spirit world and not madness. At the same time, adding personal vivid detail demonstrates that the experience is authentic and not repeated as a cultural script. This pattern is common in these ethnographic and historical accounts of hallucinations.

So is the frank identification of their nonpathological character. Dein and Littlewood^25^ interviewed 25 members of a Pentecostal church in London who said that they had heard God speak audibly. In such churches, congregants talk of “discerning” whether such a voice comes from God by asking whether the voice is in accord with scripture, gives one peace, and so forth. The anthropologists described 1 man with bipolar disorder who distinguished between God’s voice and his own experience of psychosis this way: “God says something and doesn’t force you, so you can do what you like with it … [the psychotic voices] you can’t refuse to do something when you hear them. They are very pushy.”

In such settings, people also often distinguish between unusual sensory experiences from God and those from demons. The Christian church has been intensely interested in this question, particularly during its medieval periods of great visionary activity (eg, Caciola^26^) and also throughout its history. Tracts like “The Appearance of a Spirit”^27^ describe an apparent hallucination reported to a woman in 1628 and the efforts of clerics to determine the spirit’s true nature. “Huguette [the woman who saw the spirit] is told to pay attention to its hands and its feet and its head, if may be she did not see any nails that were too long, like the talons of some bird of prey … a demon would not be able to appear for long in the guise of a man without mixing into it some wild, clawed, beaked, tailed, or horned beast.”^27^

Such culturally acceptable hallucinations are sometimes experienced by many and sometimes only by a few. Apolito^28^ identifies the former as “weak” visionaries, such as the “dancing sun” phenomenon in Europe, in which many people report that the sun behaves in peculiar, hallucination-like ways and that these apparitions indicate that Mary is at hand. An example of “stronger” visionaries are Amazonian shamans who are sometimes described by their ethnographers as reporting that they see spiritual jaguars who come and go over long periods of time and with whom they have complex conversations.^29^ Such experts are generally more practiced and sometimes describe a process of entrainment whereby over time their perceptions become more precise, more senses become involved, and the visions can occur on demand, as in the Basque visions at Ezquioga.^30^

When people report speaking with God or other supernatural agents frequently and repeatedly, anthropologists and historians have suggested that the underlying psychological mechanism is dissociation (eg, Taves^23^). They presume that the subjects have trained their attention in culturally prescribed ways, so that the shaman or possessed person who regularly hears spirits talking is best understood as going into frequent trance.

Thus, we can speak of the “cultural conditioning” of hallucination experience. Organized religions are themselves cultural systems that provide an evolving set of expectations. In Roman Catholicism, as we have seen, unusual sensory experiences have specified the location of healing shrines, established devotional practices and religious orders, and confirmed or questioned Church dogma. The embodied nature of the visions—whether the seers enter into a dissociated state, or not, and what kind of dissociation (abstraction, insensibility to physical stimuli, some kind of in-between state, catalepsy, or fits)—has varied greatly from site to site and among seers at the same site. What visionaries see and hear, when they do so, and how the experience impacts their bodies, especially when onlookers are present, all evolve over time, an indication that the visions are quite vulnerable to expectations and suggestion.

It is only in the 20th century, as Leudar and Thomas^31^ point out, that hallucinations have been described as exclusively the sign of an illness. As a result, the term “hallucination” can carry stigma. Nonetheless, events that appear technically to be hallucinations and that conform to popular expectations of the presence of God are still often reported as religious events in popular Western media.

## Hallucinations Occur at Different Rates in Different Cultural Settings

Al-Issa^32^ has suggested that Euro-American culture itself dampens the rate of hallucinations because the shared culture strives to clarify and distinguish whether a given experience is real or imaginary, and when individuals seem not to be able to make such a distinction by reporting something that seems to be a hallucination, they are likely to be labeled as out of contact with reality and therefore pathological. In contrast, he argued, many non-Western societies do not make such a rigid distinction between reality and fantasy. One might expect, then, that hallucinations would be more readily reported outside of the Western setting.

Epidemiological studies seem to support this inference. Johns et al^33^ demonstrated that reports of hallucinations in the general population varied significantly across different ethnic groups living in the United Kingdom. In this study, 5196 participants from ethnic minorities (Caribbean, Indian, African, Asian, Pakistani, Bangladeshi, and Chinese) and 2867 White UK respondents were screened for mental health problems and asked about hallucinations. Reports of hallucinations were around 2.5 times higher in the Caribbean sample (9.8%) compared with the white sample (4%). Compared with the white sample, the experience was only half as common in the South Asian sample (4% vs 2.3%).

Anthropological work certainly also demonstrates that hallucinations may suddenly increase in a social group at a particular time. For example, after the death of Menachem Schneerson—a Hasidic Rebbe believed by many of his followers to be the messiah and thus a man who would not die in an ordinary way—many followers reported seeing him.^34^ The pattern of their reports resembles the reports of seeing Jesus after his death described in the Bible: they are rare; brief; and, often, surprising mundane. Jesus appears as a gardener: the Rebbe shows up in the kitchen.

## Culture Affects the Meaning and Characteristics of Hallucinations Associated With Psychosis

Both anthropology and psychology/psychiatry have concluded that to some extent, the hallucinations associated with serious psychotic disorder are “pathoplastic,” meaning that they are shaped by local expectation and meaning. Certainly the content of hallucinations is influenced by local culture. Rural Africans are more likely to hallucinate about ancestor worship; Christians are more likely to hallucinate about Christ, Mary, and Satan. But culture seems to affect the form of hallucinations as well. Mitchell and Vierkant^35^ compared hallucinations in patients admitted in an East Texas hospital during the 1930s with those reported in patients in the same hospital in the 1980s (patients were matched for age, race, and gender distribution). They found that the hallucinations of the 1930s reflected the intense desire for material goods associated with the Great Depression, and those of the 1980s reflected the new technological tools of the 1980s. More strikingly, the command hallucinations of the 1930s were primarily benign and religious (“live right”, “lean on the Lord”), but those of the 1980s were negative and destructive (“kill yourself”, “kill your mother”). The authors suggested that the more negative commands of the later period reflected a more negative and hostile environment.

Indeed, command hallucinations seem to vary considerably. Suhail and Cochrane^36^ used case notes to compare the modalities and themes of hallucinations in 3 different groups of psychotic patients: (*a*) white British patients, (*b*) Pakistani patients living in Britain (who lived an average of 17 years in the United Kingdom), and (*c*) Pakistani patients living in Pakistan. They found that the most dissimilar pair was the white British patients and the Pakistani patients living in Pakistan. In particular, the British patients were more likely (compared to the Pakistani patients) to hear, for instance, voices commenting on behavior, personality, and actions; commands to kill self or others; and voices calling bad names. On the other hand, the Pakistani participants more often heard criticising, threatening, or insulting voices. Kent and Wahass^37^ compared the auditory hallucinations of patients with schizophrenia in Saudi Arabia and the United Kingdom and found that the Saudi Arabian patients were more likely to describe hallucinations with religious content, while the British were more likely to report a running commentary. Similarly, Okulate and Jones^38^ reported that the frequency of auditory hallucinations that were commanding, abusive, cursing, arguing, and frightening was generally lower among their Nigerian patients with schizophrenia than among patients in the United Kingdom, on the basis of findings by Nayani and David.^39^ Furthermore, in this study, voices discussing the patient in the third person were not as frequent among the Nigerian schizophrenic patients as in the UK study. It is, however, important to underline that evaluations of the 2 groups of patients were not carried out by the same team of researchers.

It also appears to be true that the rate of hallucination varies considerably in different settings. Bauer et al,^40^ using identical inclusion/exclusion criteria and identical assessment procedures, compared persons with schizophrenia in 7 different countries (Austria, Poland, Lithuania, Georgia, Pakistan, Nigeria, and Ghana). In all settings, patients were more likely to report auditory than visual hallucinations, but the 1-year prevalence rates ranged considrably: auditory hallucinations from 67% (Austria) to 91% (Ghana) and visual from 4% (Pakistan) to 54% (Ghana). Thomas et al,^41^ using identical inclusion/exclusion criteria and identical assessment procedures and comparing US patients and Indian patients, found similar results. Stompe et al^42^ examined groups of patients diagnosed with schizophrenia in the same data set later used by Bauer et al.^40^ Using discriminant analysis, they argued that between 15% and 30% of the psychotic symptomatology examined in their study was culture dependent, 16% for hallucinations specifically.

Meanwhile, Barrett^43^ found that his attempt to translate the Present State Examination from English into the Iban language failed when it came to rendering thought insertion and withdrawal. In the Iban culture, thinking arises from the heart-liver region. It is not contained in the mind, which is somehow contained in the brain—a more Western conception. Fabrega^44^ had already made this criticism of the Schneiderian first-rank symptoms: “These symptoms imply to a large extent persons are independent beings whose bodies and minds as separated from each other and function autonomously.” Barrett found that the process of making thought insertion/withdrawal questions intelligible to the Iban meant that they lost their core Schneiderian meaning.

More recently, Luhrmann et al (in press)^45^ have compared the experience of hearing voices among people with schizophrenia in San Mateo, California; Accra, Ghana; and Chennai, South India. In each setting, they interviewed 20 people with schizophrenia who were asked in detail about the phenomenology of their hallucinatory experiences, their relationships with their voices, and their experiences of their voices. They found that their American sample hated their voices, readily used the diagnostic label of schizophrenia, and could even sometimes recite diagnostic criteria. For them, the primary meaning of an external voice was being “crazy.” In general, the American sample did not treat their voices as persons, and their accounts of voice-hearing were filled with violence. Patients in Chennai and Accra, by contrast, did not use a diagnostic label, and they did not experience voice-hearing as necessarily bad. They were more likely to identify voices as people they know and more likely to describe conversational relationships with their voices. Yet, there were differences between the 2 settings. In Accra, half of the patients reported that their dominant external voice was God, that hearing God was a good experience, and (usually) that God told them to ignore the mean (or demonic) voices. In Chennai, patients were more likely to hear their kin. They often did not like the voices, but the voices usually did not tell them to kill themselves, the way the voices of the Americans often; the voices told them to get dressed, clean up, and do chores. These findings suggest that hallucinations associated with schizophrenia or serious psychotic disorder may be less caustic, on average, for persons in the non-West, compared to those in the West.

Anthropologists and psychologists have also demonstrated that kin respond to the voices heard by psychotic relatives in varying ways. Jenkins^46^ found that Mexican-Americans relatives were more likely to express tolerance and sympathy to relatives with distressing voices, while Euro-American families were more liable to generate critical or hostile responses. South Asian families too seem to respond with less “expressed emotion” than Euro-Americans.^47^ Corin and colleagues^48^ observed that, in South Asia, persons with psychosis often exhibit “positive withdrawal.” In detailed interviews of patients recently diagnosed with schizophrenia, they demonstrated that not only were patient narratives often inscribed within a religious frame but also the patients would use this religious frame of reference to support a calm inner detachment. As 1 subject remarked: “I sit patiently, quietly, and wait.” Corin et al argue that this positive withdrawal is particularly salient in Hinduism, but they found that references to it were also to be found in narratives of people interviewed by Corin in Montreal.

In sum, the evidence suggests that the voice-hearing experience is deeply shaped by local patterns of understanding the self, the mind, and the fundamental nature of reality. Jenkins^11^ captures this richness in arguing that the subjective experience of psychosis and schizophrenia provides a “paradigm case for understanding fundamental human processes” and that “hearing voices” is undeniably a fundamental self-process that is thoroughly infused with cultural meaning.

## Do the Cultural Variations of Psychotic Hallucinations Have Implications for Clinical Outcome for Those Who Struggle With Psychosis?

Studies have shown that a number of mechanisms and factors play a key role in the transition between subclinical hallucinatory experiences and clinical psychosis (see Johns et al^9^). In a population-based, longitudinal study, Krabbendam et al^49^ found that those with subclinical hallucinatory experiences at baseline who developed a depressed mood at year 1 were at increased risk of transitioning to psychotic disorder at year 3 follow-up. The authors interpret these findings in light of work showing that attributions of hallucinations as coming from a threatening, powerful, and omnipotent force will lead to feelings of helplessness and depression.^50^ If persons with psychosis experience more benign hallucinations in some cultural settings than in others, it may well be the case that the voice-hearing experience will be less clinically harmful. Indeed, both Corin and Luhrmann et al place their observations in the context of the more benign trajectory of schizophrenia in India and elsewhere outside of the West.^51^ Research with a consumer-driven movement (the Hearing Voices Movement) has found that training people who hear distressing voices to interact with their voices leads to reduced distress.^52^

It is worth bearing in mind, however, that “functional impairment” and “clinical outcome” can itself only be fully defined with regard to the cultural context. For example, the disability caused by hallucinated voices may depend a great deal on the cultural organization of work and the norms of collective toil: people who live in cultures where there is less flexibility with regard to work schedules may find themselves perhaps more impaired than those where the home-work divide is more fluid. Furthermore, there are cultural criteria for who is considered to be in need of clinical attention. In earlier decades, Schooler and Caudill^53^ found that Japanese people with schizophrenia were more likely to be identified and brought to the attention of clinical services through aggression, while British people are more likely to be identified as in need of care by the presence of hallucinations.

## Conclusion

The present review demonstrates that culture shapes hallucinations in all dimensions of the phenomena: in identification, in experience, in content, in frequency, in meaning, in the distress they elicit, and in the way in which others respond. Further, culture shapes hallucinations in both their pathological and nonpathological forms.

In a recent review of research strategies and future directions in cultural psychiatry, Kirmayer and Ben^54^ warn against the danger of reifying culture and of relying exclusively on population-level categories of nationality or ethnicity in understanding its relationship to mental ill health. We also insist that culture cannot be reduced to national or even ethnic differences and that there are complex and significant variations within cultures—religious, regional, and political. The global Hearing Voices Movement constitutes an international subculture in which hallucinatory experience is positively valued and through which individuals have been able to embrace a public identity as “voice-hearers,”^55,56^ in turn changing the ways in which they understand, relate to, and experience their voices.

Culture belongs not only to the patient but also to the professional; it plays a structural role in shaping the meaning of hallucinatory experience within a clinical setting, but no less of an important role in the context of research. Hallucinations research, like most experimental work in psychology and neuroscience, is WEIRD.^57^ That is, a majority of participants and subjects in mainstream studies live in Western, Educated, Industrialized, Rich, Democratic societies, as do the researchers who study them. This limits what is known scientifically and clinically about the ways in which hallucinations are experienced, interpreted and valued across cultures, and places renewed emphasis on the importance of ethnographic and interdisciplinary^58^ approaches, as well as on increasing the number of countries and cultural groups involved in research.

A number of issues need to be addressed in future studies. For instance, the issue of cross-cultural hallucination prevalence rates in the general (nonclinical) population has not been examined in a direct and in-depth manner. In a recent review of studies examining auditory hallucination prevalence in the general population,^10^ no such studies are reported. Further, in a worldwide cross-national (52 countries) study,^59^ highly varying prevalence rates for hallucinations among persons with psychosis across countries (0.8% in Vietnam to 31.4% in Nepal) were reported and no further analyses were carried out in order to underline any potential cross-cultural patterns. We are in need of better epidemiological work on hallucinations in both the non-clinical and the clinical populations.

There is also an important implication for epidemiological or cross-cultural assessments of the presence of hallucinations. As with the study of Nuevo et al,^59^ that used the same definition to assess for the presence of hallucination across a large number of countries, it is not clear to what extent the huge difference in prevalence is due to genuine difference in the experience of “false perception” and to what extent the difference is due to differing cultural labeling of what is relevant when discussing, “an experience of seeing visions or hearing voices that others could not see or hear.”

**Table 1.** Key Points for Future Directions

	Several important questions emerge from this overview:
1.	We still know relatively little about hallucinations cross-culturally, including prevalence rates within the nonclinical population in different cultures and within clinical populations.
2.	We also know little about cultural influences on the development of hallucinations within the life span, particularly in childhood and adolescence, for both clinical and nonclinical populations.
3.	The work reported here suggests that positively valuing psychotic hallucinations improves the patient’s experience; more work is needed to determine whether this also improves clinical outcome.
4.	The work reported here also suggests that experiencing psychotic hallucinations as a person-to-person relationship may improve the patient’s experience; again, we need more work to explore whether this improves clinical outcome.
5.	The observation that culture affects the meaning and characteristics of hallucinations suggests that clinicians might develop these observations for clinical use. Much more work remains to explore whether and how this might be done.
6.	It needs to be recognized that a clinician is also part of a culture and that the factors that affect the clinician’s interpretation of hallucinatory experiences need to be understood in making clinical judgments. More work is needed to understand this process.

Finally, findings presented in this review also have clinical implications. First, clinicians should never assume that the mere report of what seems to be a hallucination is necessarily a symptom of pathology (see Johns et al^9^). Indeed, patients who are newly bereaved may need a clinician to reassure them that hallucinations of the lost loved one are normative. Second, clinicians should take seriously the new findings, supported by this review, that hallucinatory experiences respond to cultural shaping. Thus, the clinician, in addition to providing a detailed account of the hallucinations, must also take into account a person’s cultural background when assessing and treating hallucinations. As Bentall^60^ has pointed out, failure to appreciate the cultural context may prevent clinicians from responding appropriately to the distress experienced by their patients. On the other hand, where hallucinatory experiences are culturally accepted reactions to various life events (and therefore might be quite common), the clinician may consider not intervening at all. Thus, awareness of people’s attitudes toward hallucinations (based on cultural background) may help the clinician distinguish between pathological and culturally sanctioned hallucinations.

## Funding

Wellcome Trust (098455/Z/12/Z to C.F. and A.W.).
